# Longitudinal Assessment of Cortical Excitability in Children and Adolescents With Mild Traumatic Brain Injury and Persistent Post-concussive Symptoms

**DOI:** 10.3389/fneur.2019.00451

**Published:** 2019-05-17

**Authors:** Regan King, Adam Kirton, Ephrem Zewdie, Trevor A. Seeger, Patrick Ciechanski, Karen M. Barlow

**Affiliations:** ^1^Cumming School of Medicine, University of Calgary, Calgary, AB, Canada; ^2^Hotchkiss Brain Institute, University of Calgary, Calgary, AB, Canada; ^3^Alberta Children's Hospital Research Institute, Alberta Children's Hospital, Calgary, AB, Canada; ^4^Departments of Pediatrics, Clinical Neurosciences and Community Health Sciences, Calgary, AB, Canada; ^5^Department of Pediatrics, Faculty of Medicine, The University of Queensland, Brisbane, QLD, Australia

**Keywords:** transcranial magnetic stimulation, mild traumatic brain injury, pediatrics, persistent post-concussive symptoms, cortical silent period

## Abstract

**Introduction:** Symptoms following a mild traumatic brain injury (mTBI) usually resolve quickly but may persist past 3 months in up to 15% of children. Mechanisms of mTBI recovery are poorly understood, but may involve alterations in cortical neurophysiology. Transcranial Magnetic Stimulation (TMS) can non-invasively investigate such mechanisms, but the time course of neurophysiological changes in mTBI are unknown.

**Objective/Hypothesis:** To determine the relationship between persistent post-concussive symptoms (PPCS) and altered motor cortex neurophysiology over time.

**Methods:** This was a prospective, longitudinal, controlled cohort study comparing children (8–18 years) with mTBI (symptomatic vs. asymptomatic) groups to controls. Cortical excitability was measured using TMS paradigms at 1 and 2 months post injury. The primary outcome was the cortical silent period (cSP). Secondary outcomes included short interval intracortical inhibition (SICI) and facilitation (SICF), and long-interval cortical inhibition (LICI). Generalized linear mixed model analyses were used to evaluate the effect of group and time on neurophysiological parameters.

**Results:** One hundred seven participants (median age 15.1, 57% female) including 78 (73%) with symptomatic PPCS and 29 with asymptomatic mTBI, were compared to 26 controls. Cortical inhibition (cSP and SICI) was reduced in the symptomatic group compared to asymptomatic group and tended to increase over time. Measures of cortical facilitation (SICF and ICF) were increased in the asymptomatic group and decreased over time. TMS was well tolerated with no serious adverse events.

**Conclusions:** TMS-assessed cortical excitability is altered in children following mild TBI and is dependent on recovery trajectory. Our findings support delayed return to contact sports in children even where clinical symptoms have resolved.

## Introduction

Mild traumatic brain injury (mTBI) is a significant health concern due to its frequency and the possibility that it can contribute to long-term morbidities (1–4). The pediatric population is at the highest risk of incurring a mTBI (5, 6) and ~30% of children will go on to have prolonged symptoms lasting 4 weeks or longer (7), referred to as Persistent Post-Concussive Symptoms (PPCS) (8–10). Children with PPCS often have difficulty returning to school and sport, and experience a significant negative impact on quality of life of both the child and family (11, 12).

Disturbances of cortical excitability and neurophysiology may be involved in the pathogenesis of ongoing post-concussive symptoms. Current understanding of the pathophysiology of mTBI includes a cascade of cellular damage that may result in excitotoxicity, neuronal death, and cellular energy crisis (13–15). Underlying many of these consequences are changes to ion concentration (16, 17) and thus, resting membrane potential (17). As resting membrane potential is altered, so too is the conductivity of the surrounding neurons. Alterations to membrane potential in broader cortical regions may have a global effect on cortical excitability.

Transcranial magnetic stimulation (TMS) is a useful modality for measuring neurophysiological changes after various forms of brain injury increasing our understanding of how the brain responds and changes after injury (18–20). TMS studies in adults with mTBI and PPCS have demonstrated acute alterations in primary motor cortex excitability (21–26). Findings have varied across studies but potential changes include alterations in cortical inhibitory systems such as the prolongation of the cortical silent period (cSP) (21, 22) or reduction of long interval intracortical inhibition (LICI) (23, 24, 27). Longitudinal studies examining how these alterations change over time however have been limited and none include children.

We conducted a prospective, longitudinal, controlled cohort study to determine the relationship between motor cortex excitability, and post-concussive symptoms over time in children with mTBI. We hypothesized that measurements of cortical excitability would be associated with severity of post-concussive symptoms, and that these would change over time as symptoms decreased.

## Methods

### Population

Participants were recruited in the setting of a randomized controlled trial of melatonin for the treatment of PPCS following pediatric mTBI performed in the Complex Concussion Rehabilitation Program at the Alberta Children's Hospital (28) (clinicaltrials.gov/NCT01874847). Children (aged 8–18 years) were included if they presented to the Emergency Department or Concussion Clinic with a mTBI or concussion (diagnosed by a physician *and* a history of a mechanically-induced alteration of consciousness, or change in neurological function satisfying the ACRM criteria for mTBI (7, 12, 29) and persistent PPCS at 1 month post injury. Persistent PPCS was defined as an increase in post-concussive symptoms by at least 10 points compared to baseline using the Post-Concussion Symptom Inventory (PCSI). Children were not eligible if they had a Glasgow Coma Score of <13, loss of consciousness >30 min, a previous head injury in the last 3 months, the injury was due to an assault, or if there was alcohol or illicit substance use at the time of injury. Children with a significant past medical history (e.g., epilepsy, moderate/severe developmental delay, inflammatory bowel disease) or psychiatric history (e.g., hospital admission, regular follow-up by a psychiatrist, or requiring the use of psychiatric medications) or any contraindications to TMS (30).

**Figure 1:**
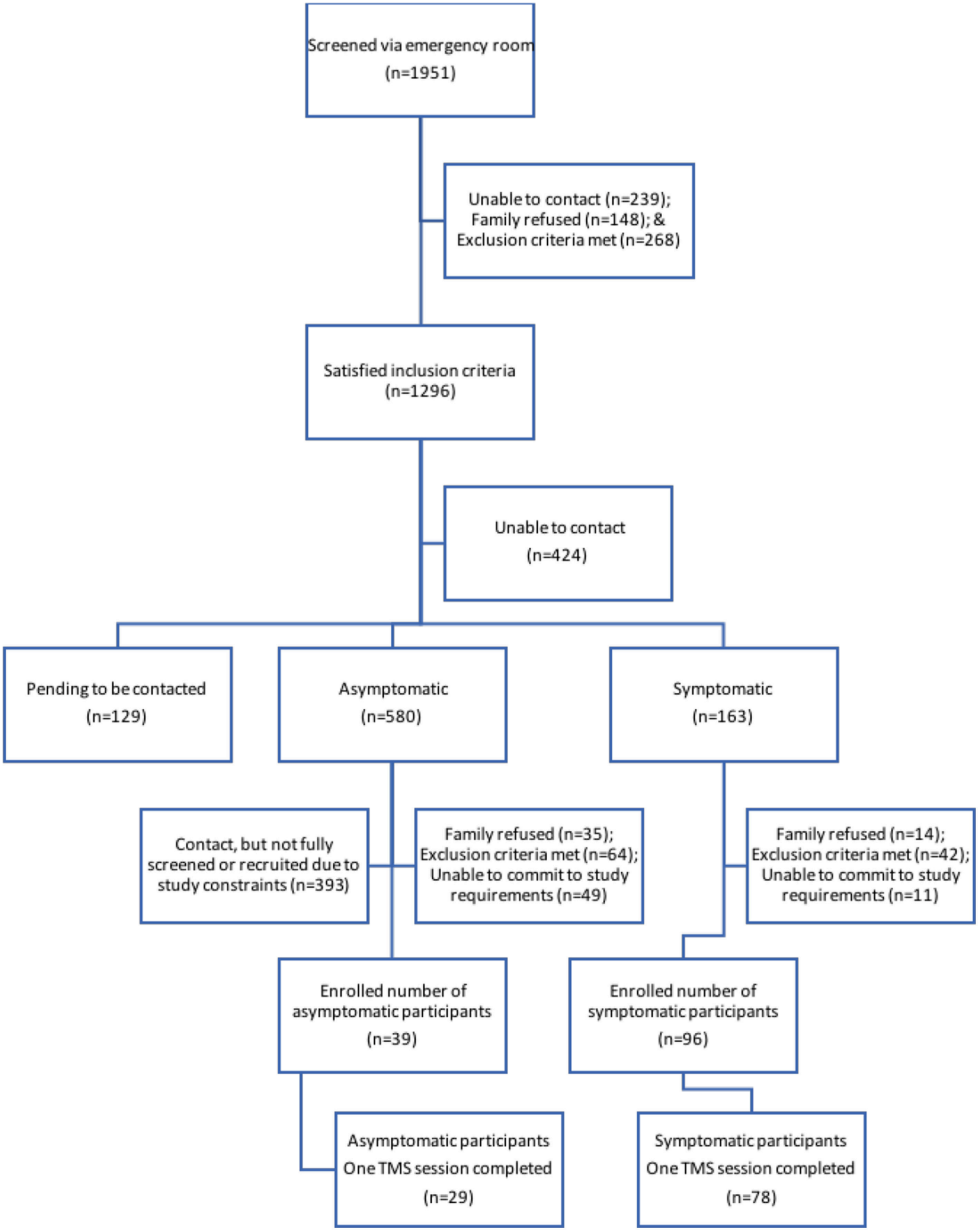
Participant recruitment flow. Schematic of individuals at each stage of the recruitment process. Final boxes show participants from each TBI group who completed the first TMS session.

Eligible families were contacted by telephone at 4 weeks post injury (Figure 1). Typically developing controls were recruited from friends and siblings of mTBI participants who satisfied all exclusion criteria including no history of TBI. Controls were recruited to maintain equal proportions of age and sex to the case population. Participants in the TBI groups were seen at 1 and 2 months post-injury and were not receiving any study medications. Each session included symptom evaluation and TMS neurophysiology. Control participants were seen at 1 month post-injury. Written informed consent was obtained from the parents of each participant, as well as verbal assent from the participant themselves. This study was approved by the University of Calgary Conjoint Health Research Ethics Board (REB13-0372).

### Clinical Outcome and Classification

The Post-Concussion Symptom Inventory (PCSI) is a validated questionnaire of 26 symptoms using a Likert scale (0–6), that provides an overall score of PPCS symptoms (range 0 to 156) (8, 31). It has four clinical domains; somatic, cognitive, affective and sleep. As PCSI symptoms are common in healthy populations, an assessment of the pre-injury PCSI score was obtained at enrollment (4 weeks post injury). Participants were designated as “symptomatic” if the PCSI score was increased by 10 or more points compared to the pre-injury PCSI score. A smaller cohort of children with clinical recovery by 4 weeks post injury was also recruited. This “asymptomatic” group had PCSI scores at or below the pre-injury score. Participants completed the PCSI at the initial TMS session (1 month post-injury), and at the follow-up TMS session at 2–3 months post injury. At follow-up, the symptomatic group was classified as “recovered” if their PCSI returned to pre-injury levels or below.

### TMS Neurophysiology Measures

Participants attended the Alberta Children's Hospital Pediatric Non-invasive Brain Stimulation Laboratory and were oriented to the TMS procedures. Participants could watch a movie of their choice while seated comfortably. Ag-AgCl electrodes (Kendall; Chicopee, MA, USA, 1.5-cm radius) were placed on first dorsal interosseous (FDI) muscles bilaterally to record surface electromyograms (EMG). Grounds were attached to a wrist band. EMG signals were amplified 1,000x and band-pass filtered from 20 to 2,000 Hz and then digitized at a rate of 5,000 Hz using CED1401 hardware and Signal 6.0 software (Cambridge Electronic Design, Cambridge, UK).

### Single-Pulse TMS

Assessment of motor cortex neurophysiology was performed by eliciting motor evoked potentials (MEPs) in the dominant FDI muscle using single pulse TMS of contralateral primary motor cortex (M1). TMS was performed with a figure-of-eight coil (70 mm) connected to a Magstim Bistim^2^ stimulator (Magstim; Dyfed, UK) which induced posterior-anterior currents in the dominant motor cortex while recording bimanual FDI. The optimal location or ‘hotspot' that produced the largest and most consistent MEP was determined using suprathreshold intensities, which were then reduced. The hotspot was marked on a standard T1 MRI using neuronavigation (Brainsight 2, Rogue Research, Montreal, CA) and used for all further testing. For active TMS trials, FDI contraction was held at 20 or 50% maximum voluntary contraction with continuous visual feedback via an oscilloscope (GDS-1022, GwINSTEK, Taiwan) with the full-wave EMG rectified and smoothed (100 ms time constant, Neurolog NL703EMG Integrator, Digitimer UK).

Following localization of the motor hotspot, the resting motor threshold (RMT) was determined per published standards (32) as the minimum stimulator intensity (percentage of maximum stimulator output, MSO) required to elicit >50 μV FDI motor-evoked potentials (MEP) in 5/10 consecutive trials. MEP were recorded by Signal software and imported into MATLAB R2014b (Mathworks, Inc., Natick MA) for scripted analysis. The EMG from each paradigm was manually inspected for artifacts and pre-stimulation muscle activation, with poor quality frames removed. EMG traces were blinding prior to manual analysis to eliminate experimenter bias. All MEP analyses were performed using Matlab scripts developed by our team.

### Single Pulse TMS Outcomes

#### Stimulus Response Curve (SRC)

Ten single pulse TMS stimulations at each of six intensities (100–150% RMT, in steps of 10%) were delivered in random order to produce recruitment curves. MEP were recorded bilaterally from FDI muscles. Recruitment curves were analyzed by measuring peak to peak amplitude of 10 MEP from six intensities (100–150% RMT, in steps of 10%) then averaged to produce a sigmoidal input/output curve.

#### Cortical Silent Period (cSP)

Fifteen suprathreshold stimuli were applied with a three second separation while the dominant hand was contracted at 20% maximum voluntary contraction. The silent period was defined as a continuous disrupted EMG waveform beginning after the end of MEP waveform, continuing until the return of the background EMG waveform.

#### Ipsilateral Silent Period (iSP)

Ten suprathreshold (120% RMT) TMS stimuli were applied during 50% maximal voluntary contraction in the non-dominant hand (ipsilateral to the stimulated M1). Silent periods were measured synonymously to cSPs. The start of the silent period was manually determined as the point at which the EMG trace dropped below 25% of the background. The end of the silent period was determined when the EMG trace returned above 25% of the normal background. Silent periods were averaged to produce a mean silent period duration.

### Paired Pulse TMS Outcomes

Paired-pulse TMS used two stimulators (Bistim^2^ and 200^2^ Magstim, UK) connected by an adaptor. Paired pulses were separated by an interstimulus interval (ISI) between an initial conditioning stimulus (CS, 80% RMT), followed by a test stimulus (TS, 120% RMT). Paired pulse outputs were expressed as a ratio of CS:TS, with >1 indicating facilitation and <1 indicating inhibition.

#### Short Interval Intracortical Inhibition/Intracortical Facilitation (SICI/ICF)

*Ten* unconditioned TS were randomly intermixed with 10 paired stimulation each at 10 ms ISI for ICF and 2 ms ISI for SICI (total of 30 stimulations). *Long Interval Intracortical Inhibition (LICI):* Here, the CS and TS were separated by a 100 ms ISI. Ten CS-TS pairs were randomized with ten TS alone. *Short Interval Intracortical Facilitation (SICF*): Ten unconditioned and 10 conditioned stimuli at each of three separate ISIs (1.5, 2.6, 4.3 ms) were delivered in random order (40 stimulations in total).

### Safety and Tolerability

After each session, participants completed a previously developed pediatric TMS tolerability questionnaire (33) that documented the presence and severity of five common potential side effects (headache, unpleasant tingling, neck pain, nausea, and light-headedness) (34). Participants were also asked to rank their TMS experience against 7 other common childhood experiences.

### Statistical Analyses

The sample size was estimated as 24 per group using the cSP data from Miller et al. (23). Demographic characteristics were compared between experimental and control groups using student *t*-tests and Chi-square tests. Between group contrasts of SRC data indicated no significant changes in activation across sessions (using a two-way ANOVA). One sample *t*-tests were performed to determine the presence of inhibition or facilitation, as per our stipulated paired pulse ratio criterion. Partial correlations were used to analyse the relationship between change in cortical excitability and PCSS symptoms controlling for the PCSI pre-injury scores. We used generalized linear mixed models (GLMM) were used to analyse the relationships between Group and Time characteristics in a linear mode (cSP and iSP) and log-normal mode (SICI, LICI, SICF, and ICF) in our neurophysiological data. Time and group were fitted as fixed factors and participant as a random factor. Where there was an effect of time, GLMM in logistic mode (with Satterthwaite approximation) was used to analyze the relationship between recovery and cortical excitability change in the symptomatic group. Analyses were performed using SigmaPlot (version 13.0) and SPSS (version 24) software.

## Results

### Population

Details of participant recruitment are shown in Figure 1. Population demographics are summarized in Table 1. One hundred and sixty-one participants were enrolled, and 133 had measurable and complete stimulus response curves allowing them to complete the entire study protocol. The final experimental sample consisted of 107 participants (78 symptomatic and 29 asymptomatic) with a median age of 15.1 (range: 9.0–18.0 years, 57% female). A smaller experimental population completed a second session of TMS (see Table 1). The healthy control population included 26 participants (median age = 14.6 years, range: 9.9–18.0 years, 54% female). The median age of the experimental groups was comparable, as was the ratio of males to females.

**Table 1:** Population demographics. Demographic characteristics and recovery status^a^.

	**Control**	**Asymptomatic**	**Symptomatic**
N at Session 1	26	29	78
N at Session 2	-	9	54
Age ± SD, Range	14.6 ± 3, 9–18	14.2 ± 2, 9–17	15.2 ± 2, 9–17
Gender, F%	54	48	60
Handedness, R%	88	86	90
Cause of Injury %			
• Sport		82	71
• Fall		12	7
• MVA		3	10
• Other		3	12
PCSI Δ 1mo (median)	-	1	28
PCSI Δ 2mo (median)	-	4	3
Cognitive Domain Score	0.55 (1.2)	0.93 (1.6)	11.8 (8.1)
Physical Domain Score	1.6 (2.4)	2.3 (3.4)	16.2 (10.4)
Emotional Domain Score	0.75 (1.3)	0.65 (1.5)	7.2 (6.2)
Fatigue Domain Score	0.85 (1.0)	0.9 (1.8)	5.4 (4.1)

a *TBI participants were divided into 2 groups; symptomatic and asymptomatic. Mean age was expressed in years, ± standard deviation. Dominant handedness was reported as % right hand dominant. PCSI scores were expressed as the median change from baseline. Asterisk indicates significantly different from controls*.

### Single Pulse TMS

#### Stimulus Response Curves

**Figure 2:**
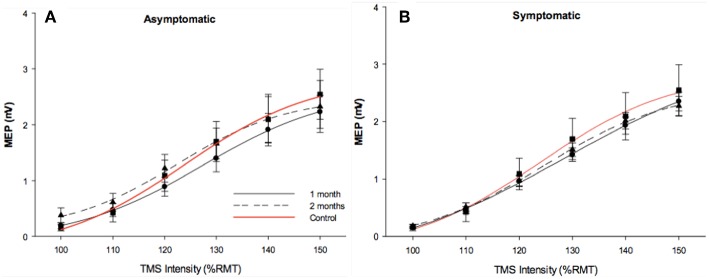
Stimulus response curves. Stimulus response curves from dominant FDI muscle in 1 month post injury (solid black line), 2 months post injury (dotted black line). Control data (red line) was included in each figure as a reference. Longitudinal asymptomatic data shown in plot A (*n* = 29), symptomatic data in plot B (*n* = 78). Group means were comparable across session.

Resting SRCs were obtained from all participants across each experimental group. Sigmoidal curves are shown in Figure 2. Responsiveness to increased TMS intensity were similar across groups at the initial measurement (*p* = 0.5), and at follow-up (*p* = 0.9).

#### Cortical Silent Periods

CSP was measured in a total of 124 participants (29 asymptomatic, 69 symptomatic, 26 control). cSP did not differ between groups overall, [*F*_(2, 121)_ = 0.281, *p* = 0.281]. Mean MEP amplitude, which may effect cSP, did not differ between groups. There was no effect of age or sex on cSP change over time. Generalized linear mixed-effects model demonstrated shorter cSP durations (18.84 (SD 2.82) ms) in the symptomatic group compared to asymptomatic group at the initial timepoint [*F*_(1, 167)_ = 4.838, *p* = 0.029], with a trend for cSP duration to increase over time (11.36 (SD 2.60) ms; [*F*_(1, 84.51)_ = 3.27, *p* = 0.074]. There was significant subject heterogeneity (random intercept) accounting for 22% of the variance (95% CIs: 12.14, 36.18). Recovery in the symptomatic group at 2–3 months post injury could be predicted by cSP duration at 1 month (OR 1.029, *p* = 0.049) and change in cSP over time (OR.984, *p* = 0.006). Although the overall model was significant (LR Chi (2) = 9.78, *p* < 0.001), the effect size was small.

#### Ipsilateral Silent Periods

**Figure 3:**
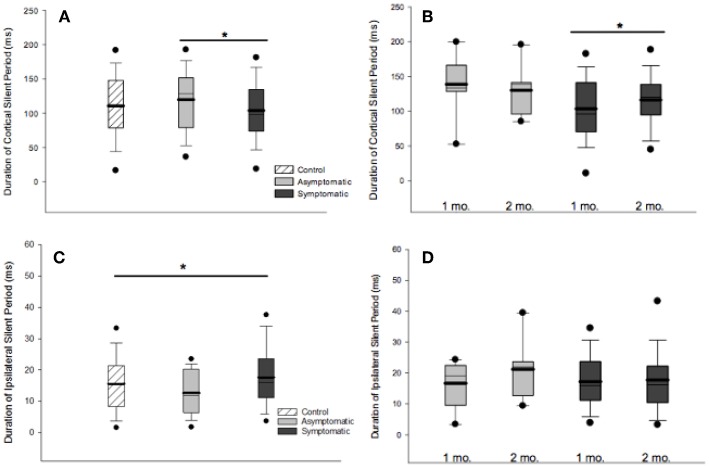
Cortical and ipsilateral silent periods. **(A)** Comparison of cSP duration across groups at 1 month post injury found shorter cSP duration in the symptomatic compared to the asymptomatic group. **(B)** cSP durations increased over time in the symptomatic group at 1 month than 2 months post injury [*F*_(1, 167)_ = 4.838, *p* = 0.029]. **(C)** iSP durations differed across groups at 1 month post injury, [*F*_(2, 119)_ = 3.828, *p* = 0.0240]. **(D)** Generalized linear model mixed-effects modeling demonstrated longer iSP durations in the symptomatic group [F_(1, 112)_ = 4.052, *p* = 0.046] but no effect of time [*F*_(1, 76)_ = 0.977, *p* = 0.324]. For all boxplots, thick line is mean, thin line is median, edges are quartiles, and whiskers are 5 and 95th percentiles. ^*^p < 0.05.

ISP was measured in a total of 123 participants (28 asymptomatic, 69 symptomatic, 26 healthy controls). Significant differences in iSP across all groups were observed [*F*_(2, 119)_ = 3.828, *p* = 0.024]: Symptomatic EM = 18.044 (95% CI: 16.092, 19.996), Asymptomatic EM = 14.150 (95% CIs: 10.823, 17.478), Control EM = 16.263, (95% CIs 12.433, 20.093). Generalized linear mixed-effects modeling demonstrated longer iSP durations in the symptomatic group [*F*_(1, 112)_ = 4.052, *p* = 0.046] but no effect of time [*F*_(1, 76)_ = 0.977, *p* = 0.324] (Figures 3C, D).

### Paired Pulse TMS

#### Short Interval Intracortical Inhibition

**Figure 4:**
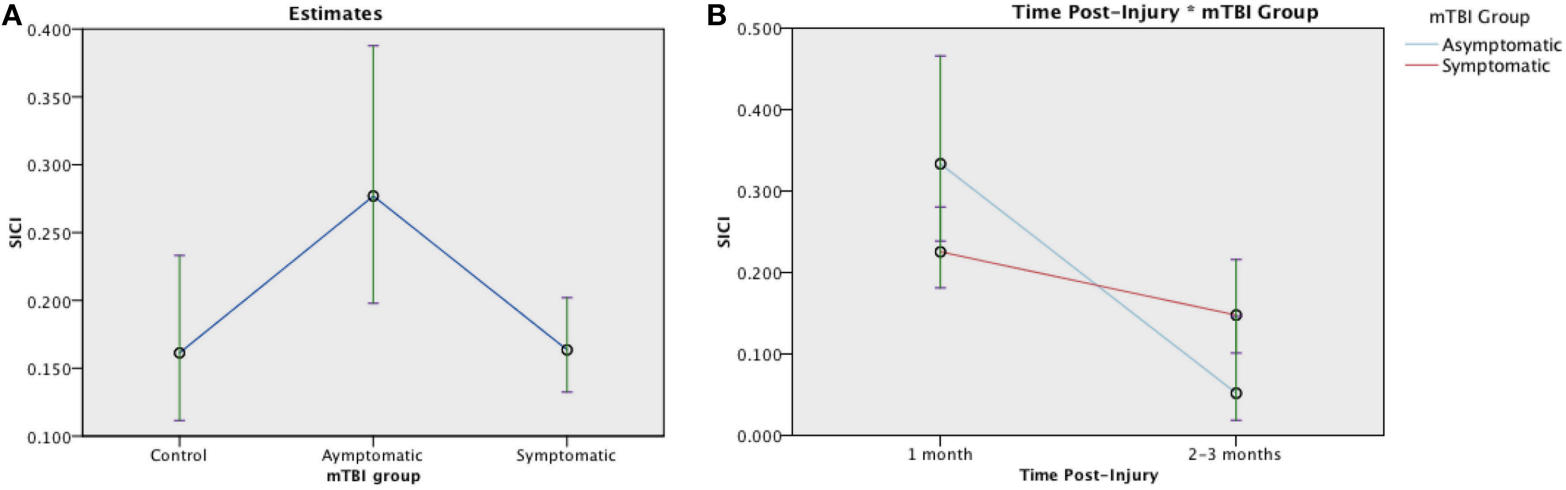
Estimated means charts for SICI. SICI is expressed as a ratio of conditioning stimulus (CS, 80% RMT) to test stimulus (TS, 120% RMT) at interstimulus interval of 2 ms. Values <1 indicate inhibition. **(A)** Significant differences were found in SICI between groups at 1 month post injury, [*F*_(2, 128)_ = 3.752, *p* = 0.026]. **(B)** Generalized linear mixed model analysis demonstrated a significant effect of Time and Time × Group interaction on SICI in mTBI groups corrected model [*F*_(3, 179)_ = 8.135, *p* < 0.001]. Although both the symptomatic (red) and asymptomatic (blue) groups show decreasing SICI over time, the trajectory is steeper in the asymptomatic group. The error bars represent 95% confidence intervals of the estimated means.

SICI was evaluated in 131 participants (30 asymptomatic, 76 symptomatic, 25 control). An average inhibitory effect consistent with SICI was present in all groups [*t*_(25)_ = 4.372, *p* < 0.001]. Generalized linear mixed-effect modeling revealed significant differences in SICI at 1 month post injury between groups, [*F*_(2, 128)_ = 3.752, *p* = 0.026]: Symptomatic EM = 0.164 (95% CI:0.132, 0.202), Asymptomatic EM = 0.277 (95% CIs:0.198, 0.388), Control EM = 0.161, (95% CIs.112, 0.233) (Figure 4A). Change over time was analyzed in the TBI groups. Here, the effect of Group was not significant [*F*_(1, 179)_ = 1.005, *p* = 0.317] but there was a significant effect of Time post injury [F_(1, 179)_ = 18.746, *p* < 0.001] and a significant Group × Time interaction [*F*_(1, 179)_ = 18.746, *p* < 0.007] (Figure 4B). Logistic regression, however, failed to demonstrate a relationship between recovery and change in SICI over time in the symptomatic group (LR chi(2) = 0.59, *p* = 0.744).

#### Long Interval Intracortical Inhibition

LICI was evaluated in 128 participants (30 asymptomatic, 73 symptomatic, 25 control). A significant inhibitory effect consistent with LICI was present in each group [*t*_(25)_ = 4.372, *p* < 0.001]. There were no significant difference in LICI between groups, [*F*_(2, 125)_ = 0.678, *p* = 0.509]. Over time, GLMM demonstrated no effect of mTBI group [*F*_(1, 174)_ = 1.453, *p* = 0.230] or Time post-injury [*F*_(1, 174)_ = 0.623, *p* = 0.431].

#### Intracortical Facilitation

**Figure 5:**
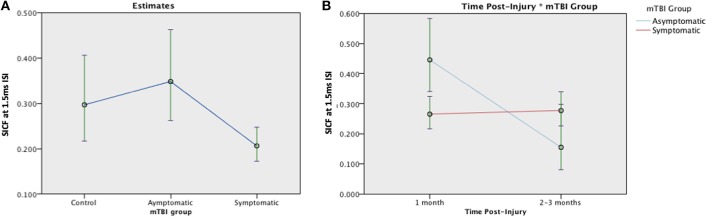
Estimated means charts for SICF effect at 1.5 ms ISI. **(A)** The SICF effect differed significantly across groups [*F*_(2, 117)_ = 5.46, *p* = 0.005]. **(B)** There was a significant effect of Time and a Group × Time interaction effect, corrected model [*F*_(3, 168)_ = 6.058, *p* = 0.001]. The error bars represent 95% confidence intervals of the estimated means.

SICF was measured in 120 participants (28 asymptomatic, 69 symptomatic, 23 control). The SICF effect was present at both ISI in the control group at 1 month (ISI 1.5 ms, *p* = 0.05; 4.3ms, *p* = 0.02) (Figure 5). There was a significant effect of Group [*F*_(2, 117)_ = 5.46, *p* = 0.005] on the SICF effect: Asymptomatic estimated mean (EM) = 0.348 (95%CI:0.262, 0.463), symptomatic EM = 0.206 (95% CI:0.172, 0.247), and control EM = 0.297 (95% CI:0.217, 0.406), see Figure 5. Change was analyzed using a generalized linear mixed effects model, there was no effect of mTBI Group [*F*_(1, 168)_ = 0.021, *p* = 0.885] on the SICF at 1.5 ms ISI effect but a significant effect of Time [*F*_(1, 168)_ = 9.064, *p* = 0.003]. There was a significant Group × Time interaction effect, [*F*_(1, 169)_ = 10.719, *p* = 0.001]. There were similar effects of Group and Group × Time on SICF at 2.6ms and 4.3 ms (data not shown). Change in SICF at 1.5 ms ISI however was not related to recovery in the symptomatic group (*B* = −0.959, *p* = 0.423).

**Figure 6:**
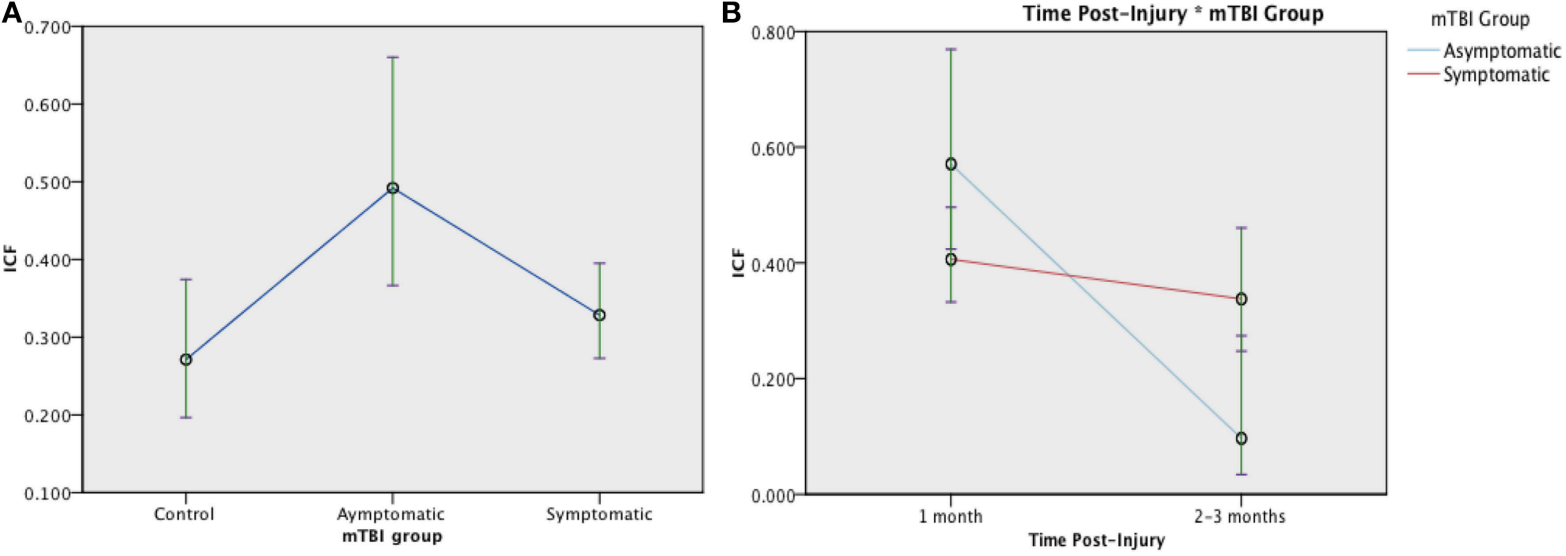
Estimated means charts for ICF. ICF is expressed as a ratio of conditioning stimulus (CS, 80% RMT) to test stimulus (TS, 120% RMT) at interstimulus interval of 10 ms. **(A)** There was a significant effect of Group at 1 month post injury, [*F*_(2, 128)_ = 4.076, *p* = 0.019]; **(B)** There was a significant effect of Time and Time × Group interaction on ICF [corrected model *F*_(3, 179)_ = 9.154, *p* < 0.001]. Although both the symptomatic (red) and asymptomatic (blue) groups show decreasing ICF over time, the trajectory is steeper in the asymptomatic group. The error bars represent 95% confidence intervals of the estimated means.

A typical range of facilitation was observed in all groups using the 2 ms ICF paradigm (*n* = 131). There was a significant effect of Group at 1 month post injury, [*F*_(2, 128)_ = 4.076, *p* = 0.019]: Symptomatic EM = 0.328 (95% CI:0.273, 0.395), Asymptomatic estimated mean (EM) = 0.492 (95%CI:0.367, 0.660), and control EM = 0.271 (95% CI:0.196, 0.374), see Figure 6. When change was analyzed with GLMM, there was no longer an effect of Group [*F*_(1, 179)_ = 0.785, *p* = 0.377], but ICF decreased significantly over time [*F*_(1, 179)_ = 11.826, *p* = 0.001] with a Time × Group interaction effect [*F*_(1, 179)_ = 6.347, *p* = 0.013]. (Figure 6). Change in ICF in the symptomatic group however was not related to recovery in the symptomatic group (*B* = 1.226, *p* = 0.072).

#### Tolerability

No serious adverse events were reported. TMS was well-tolerated with sessional tolerability measures summarized in Table 2. Mild headache was reported in 13% of participants with a higher proportion in the symptomatic group (27%). This difference at 1 month did not persist to the session completed at 2-months post-injury.

**Table 2:** Tolerability. Longitudinal tolerability data^b^.

	**Healthy**	**Asymptomatic**	**Symptomatic**	***X*^**2**^**	***P* (2)**
**Headache**					
1mo	1 (4)	4 (12)	21 (27)	8.1	0.02
2mo	–	4 (30)	9 (12)	0.6	0.4
**Neck Pain**					
1mo	1 (4)	5 (15)	11 (14)	2.2	0.3
2mo	–	1 (8)	17 (24)	0.9	0.3
**Tingling**					
1mo	0 (0)	5 (15)	11 (14)	4.3	0.1
2mo	–	1 (8)	10 (14)	0.03	0.9
**Nausea**					
1mo	0 (0)	2 (6)	4 (5)	1.5	0.5
2mo	–	2 (15)	3 (4)	0.9	0.3
**Lightheaded**					
1mo	2 (8)	1 (3)	8 (10)	1.6	0.4
2mo	–	2 (15)	3 (4)	0.9	0.3

b *Tolerability values were expressed as the total number of participants in each group that reported each side effect. The number in parentheses represented the corresponding percentage of the group*.

## Discussion

In this study, we have characterized multiple aspects of motor cortex neurophysiological change in children recovering from mTBI and PPCS. The cSP is shortened at 1 month in the symptomatic children and increases over time. Overall, parameters associated with cortical inhibition (cSP and SICI) were more likely to be relatively increased in asymptomatic children. Conversely, parameters associated with cortical excitation/facilitation (SICF and ICF) were decreased in symptomatic children. Taken together, these findings suggest that longitudinal neurophysiological measurements, via TMS, over the motor cortex suggest a demonstrable change in cortical excitability between symptomatic and asymptomatic PPCS groups. Our findings substantially add to current understanding of neurophysiological alterations that occur following a pediatric mTBI, and support the safety and tolerability of TMS in this population.

There is a paucity of research regarding the neurophysiological changes occurring in PPCS in adults and especially children. Increased cSP duration has been reported previously in adults with mTBI (21, 22, 24, 35). The only other longitudinal TMS study following 15 adults with acute mTBI reported increased cSP durations at 72 h post-injury and persisted beyond 8 weeks post injury (23). Although not reported specifically, these adults seem to have experienced normal recovery trajectories with symptom scores similar to controls by 1-month post-injury i.e., similar to our asymptomatic group (21, 27, 36). Tremblay et al. (25) Other research in asymptomatic adults with a history of mTBI found increased cSP durations from 9 months to 30 years post-injury (19, 21, 23, 25). Interestingly, the cSP duration increased over time in our symptomatic group i.e., becoming similar to the asymptomatic group. However, this was not significantly related to clinical PPCS scores or recovery. Further longitudinal studies are required to elucidate the trajectory of cSP duration over time.

Short-interval intracortical inhibition was increased in the asymptomatic group and changed significantly over time. This finding is somewhat in keeping with the changes in cSP reported above and support the cortical origin of the neurophysiological changes. Indeed, inhibitory interneurons have been shown to be particularly vulnerable to injury in TBI (37). In contrast, long-interval intracortical inhibition was not significantly different between groups in our study. Previous research in smaller samples of asymptomatic athletes with concussion has reported both enhanced LICI (25) and no significant differences to controls (25). LICI and cSP have often been thought to operate by similar cellular and neurotransmitter mechanisms including alterations in GABA_b_ receptor-mediated processes (38, 39). However, studies in other populations of acquired brain injury and neurodegeneration have suggested a dissociation between the neuronal systems reflected in these two paradigms (38, 39). Although Tremblay et al. report possible metabolic imbalances between GABA and glutamate concentrations in previously concussed athletes using MR spectroscopy (25), further well-powered research is required using multimodal imaging to tease out whether this occurs in mild TBI.

**Table 3:** Summary of results.

	**Control**	**Asymptomatic**	**Symptomatic**
N at Session 1	20	32	72
N at Session 2	-	12	71
**cSP**			
Mean (SD) T1	110.52 (8.9)	120.38 (8.4)^+^	103.24 (5.4)^+^
Mean (SD) T2	-	134.16 (11.87)	113.55 (5.4)
**iSP**			
Mean (SD) T1	15.62 (1.9)	14.15 (1.7)^*^^+^	18.04 (0.9)^*^^+^
Mean (SD) T2	-	19.29 (3.3)	18.12 (1.3)
**LICI**			
Mean (SD) T1	0.06 (0.01)	0.08 (0.008)	0.06 (0.008)
Mean (SD) T2	-	0.05 (0.06)	0.15 (0.03)
**SICI**			
Mean (SD) T1	0.16 (0.03)	0.28 (0.05)^*^^+^	0.16 (0.02)^*^^+^
Mean (SD) T2	-	0.0.5 (0.03)	0.15 (0.03)
**SICF**			
Mean (SD) T1	0.30 (0.05)	0.35 (0.05)	0.21 (0.02)
Mean (SD) T2	-	0.16 (0.05)	0.28 (0.03)
**ICF**			
Mean (SD) T1	0.27 (0.04)	0.49 (0.07)^*^^+^	0.33 (0.03)^*^^+^
Mean (SD) T2	-	0.1 (0.05)	0.34 (0.05)

* indicates significantly different from control group.

+ *indicates significantly different between symptomatic and asymptomatic groups*.

Our previous pilot study examining cortical excitability at 1 month post injury found reduced LICI in children with persistent symptoms following mTBI (40). The current study follows on from this. It is likely that the main reason for the discrepant finding is likely due to sample size, especially as logarithmic transformation can underestimate the variability in the sample. This is particular important as children with mTBI have greater variability in motor cortex neurophysiology (33). This has been addressed in our current larger study by employing generalized linear mixed effect modeling in order to take into account the between and within subject variability and maximize sample size.

In keeping with changes in cortical inhibition, we also found significant differences in intracortical facilitation (ICF and SICF). This is the first study to examine SICF in pediatric mTBI. Increased ICF was present in the asymptomatic group, and decreased SICF in the symptomatic group with both groups changing toward “normal” over time. Similar SICF results were found across the different ISI intervals which supports the legitimacy of this finding. SICF is also suggested to be mediated by GABAergic systems, with increased GABA activity resulting in reduced SICF (41). Loss of facilitation in the asymptomatic group, combined with prolonged cSP in the same population, might suggest underlying differences between the normal behavior of GABA in the motor cortex compared with those of controls or symptomatic children. Interestingly, the rate of change was greatest in the asymptomatic group. This finding supports other recent literature suggesting that there is ongoing cerebral recovery despite resolution of clinical symptoms (42, 43). The change in ICF and SICF in the symptomatic group did not predict recovery of symptoms. This may be due to the slow rate of change in ICF and SICF in the symptomatic group during this time period. The next step to better elucidate these underlying mechanisms might be to quantify GABA levels using neuroimaging methodology such as MR spectroscopy. This technique would allow quantification of GABA release patterns over time, which may be involved in mediating changes in cortical neurophysiology (44).

Transcallosal inhibition was measured using iSP. Transcallosal tracts have been shown to be particularly sensitive to damage in TBI (45, 46). iSP values were significantly different between groups with the asymptomatic group having decreased transcallosal inhibition. Measurement of iSP in children has been infrequent and somewhat inconsistent, in part due to differences in methodology. We used 120% RMT, based on methods of 40, who found iSP to be associated with paired-pulse TMS measures of interhemispheric inhibition and motor performance in a pediatric population (47). Transcallosal injury provides a complex research target due to the elaborate circuitry involved. Future research into transcallosal injury using iSP, IHI and imaging measures of both structural and functional connectivity might better elucidate any alteration of interhemispheric interactions in children with mTBI.

Significant limitations of our study should be considered. Groups were comparable in age- and gender but a significant proportion lacked repeated measures, this decreased the power in the longitudinal component of our study (especially in the asymptomatic group) although this was somewhat limited by our statistical approach. Nevertheless, the intersession variability of TMS neurophysiology remains a significant challenge in this type of research (48). Pre-injury neurophysiological data was not available in our participants. Although it is it is possible that the TMS parameter changes could have been present pre-injury, the rate of change observed over time would suggest otherwise. No differences between the SRC measures of groups, or of sessions, were observed. Our population did not include children younger than 8 years of age as younger children often have thresholds too high to stimulate at 150% RMT. Our findings suggest that the cortical excitability properties reflected by SRC are similar across participants and perhaps not altered by mTBI. This finding suggests that SRC may not be sensitive to such changes and is perhaps a paradigm that is less likely to yield interesting information in future studies.

Despite these limitations, the power of our study was much higher than the previous work in this field. Additional strengths of our study included well-standardized TMS methodologies designed to deliver stimulus intensity based on each individual participants RMT, as well as a participant group displaying a broad range of PPCS phenotypes. TMS has been safe and well tolerated all participants, with no adverse events (35).

## Conclusions

In summary, children with different recovery trajectories after mTBI show significant and complex alterations in TMS measures of cortical excitability which change during recovery. Cortical inhibition is increased in children who have early recovery whereas cortical excitation is decreased in children with persistent symptoms. Motor cortex neurophysiology changed significantly over time. These finding suggests that there is ongoing cerebral recovery at 1-month post-injury even where there is resolution of clinical symptoms. Further well-powered longitudinal studies of pediatric TBI can help to inform our knowledge and monitor neurophysiological recovery following pediatric mTBI.

## Data Availability

The datasets generated for this study are available on request to the corresponding author.

## Ethics Statement

Ethical clearance was granted by the University of Calgary Conjoint Health Research Ethics Board (REB13-0372).

## Author Contributions

KB and AK conceived study design. RK and KB performed participant recruitment. RK, EZ, PC, and TS performed data collection. RK, KB, EZ, PC, and TS performed data analysis and drafting the manuscript. RK, AK, EZ, PC, TS, and KB revised the manuscript. KB and AK obtained funding.

### Conflict of Interest Statement

The authors declare that the research was conducted in the absence of any commercial or financial relationships that could be construed as a potential conflict of interest.
